# Your Regulatory T Cells Are What You Eat: How Diet and Gut Microbiota Affect Regulatory T Cell Development

**DOI:** 10.3389/fnut.2022.878382

**Published:** 2022-04-20

**Authors:** Jian Tan, Jemma Taitz, Shir Ming Sun, Lachlan Langford, Duan Ni, Laurence Macia

**Affiliations:** ^1^Charles Perkins Centre, The University of Sydney, Sydney, NSW, Australia; ^2^School of Medical Sciences, Faculty of Medicine and Health, The University of Sydney, Sydney, NSW, Australia; ^3^Sydney Cytometry, The University of Sydney and The Centenary Institute, Sydney, NSW, Australia

**Keywords:** regulatory T cell, tolerance, nutrition, nutritional immunology, gut microbiota, thymopoiesis

## Abstract

Modern industrial practices have transformed the human diet over the last century, increasing the consumption of processed foods. Dietary imbalance of macro- and micro-nutrients and excessive caloric intake represent significant risk factors for various inflammatory disorders. Increased ingestion of food additives, residual contaminants from agricultural practices, food processing, and packaging can also contribute deleteriously to disease development. One common hallmark of inflammatory disorders, such as autoimmunity and allergies, is the defect in anti-inflammatory regulatory T cell (Treg) development and/or function. Treg represent a highly heterogeneous population of immunosuppressive immune cells contributing to peripheral tolerance. Tregs either develop in the thymus from autoreactive thymocytes, or in the periphery, from naïve CD4^+^ T cells, in response to environmental antigens and cues. Accumulating evidence demonstrates that various dietary factors can directly regulate Treg development. These dietary factors can also indirectly modulate Treg differentiation by altering the gut microbiota composition and thus the production of bacterial metabolites. This review provides an overview of Treg ontogeny, both thymic and peripherally differentiated, and highlights how diet and gut microbiota can regulate Treg development and function.

## Introduction

Breakdown of immune tolerance is a feature of many non-communicable diseases. Regulatory T cells (Tregs) are a subset of anti-inflammatory CD4^+^ T cells defined by their expression of the transcription factor FoxP3 (1). Treg mediate immune tolerance, and their deficiency, or a defect in their function, leads to anomalous immune responses to innocuous food and commensal bacteria-derived antigens, as well as to self-antigens (2, 3). Consequently, this results in various inflammatory disorders, including autoimmunity and allergies. These diseases are commonly associated with a western lifestyle. Diet appears to be one of the most influential environmental factors regulating Treg biology. Macronutrients, micronutrients, caloric content, and food additives were all shown to influence the ontogeny and function of Treg (4, 5). Diet also alters the gut microbiota, subsequently regulating Treg biology (6). Treg have either a central origin and differentiate in the thymus during the T cell selection process, referred to as thymic-derived Treg (tTreg), or a peripheral origin where they differentiate from naïve T cells in response to environmental antigens and cues such as dietary or bacterial metabolites, referred to as peripheral Tregs (pTregs) (7). tTreg and pTreg vary in their ontogeny, are regulated differently, and serve different functions, ensuring their complementary roles in immune tolerance and homeostasis. For example, pTregs are better suppressors of allergic airway diseases than tTregs of the same specificity (8), while tTregs are more efficient at suppressing experimental autoimmune encephalomyelitis (9). The difference in response likely relates to their differential expression of regulatory markers such as CTLA-4, PD-1, and their different capacity for cytokine production. This review will discuss the intrinsic and extrinsic factors that regulate Treg differentiation, highlighting the multiple avenues to promote and maintain Treg through dietary intervention. A glossary of key terms used in this review is presented in Table 1.

**Table 1:** Glossary of key terms.

CD4	CD4 is a co-receptor expressed by CD4^+^ T cells. Following engagement of the T cell receptor/CD4 complex, naïve CD4^+^ T cells differentiate into various CD4 helper T cells, including regulatory T cells involved in suppressing immune responses and inflammation. Other helper T cells, such as Th17 cells, typically mediate proinflammatory immune response.
CD28	CD28 is a co-stimulatory receptor expressed by T cells. Activation of CD28 by its ligand CD80 or CD86 is necessary to provide the co-stimulation required for effective T cell receptor signaling and activation of T cells.
CD80/86	Both CD80 and CD86 are ligands for CD28 and is expressed mostly by antigen-presenting cells. Both CD80 and CD86 can also bind to CTLA-4, which instead attenuates the T cell receptor signaling response.
CD25	High-affinity receptor for IL-2. CD25 is highly expressed by Treg and its activation by IL-2 is crucial for the maintenance and survival of Treg.
CD103	Also known as Integrin alpha-E, CD103 is a receptor involved in cell homing and adhesion via its binding to its ligand E-cadherin. CD103 is expressed by specialized subset of mucosal dendritic cells (known as CD103^+^ dendritic cells) that promotes the differentiation of regulatory T cells.
CD69	CD69 expression is upregulated following the activation of T cells. CD69 expression promotes Treg differentiation and also enhances their suppressive by promoting IL-2 and TGF-β production (10)
CD14	CD14 is expressed mostly by macrophages and act as a co-receptor for the detection of bacterial lipopolysaccharide (LPS) alongside Toll-like receptor 4 (TLR4) and MD-2.
T cell receptor (TCR)	T cell receptor are expressed by all T cells and recognize specific antigen (typically peptides) presented by antigen-presenting cells on major histocompatibility complex (MHC) molecules. Engagement of TCR with peptide-MHC molecule leads to the activation of the T cell.
Thymocytes	T cell lineage committed progenitors that develops into mature naïve CD4^+^ or CD8^+^ T cells following negative and positive selection in the thymus.
Antigen-presenting cells (APC)	Antigen-presenting cells present antigens loaded on MHC molecules for presentation to naïve T cells and are thus involved in the initiation of an adaptive immune response.
Dendritic cells (DC)	Dendritic cells are a major subset of professional antigen-presenting cells
Medullary thymic epithelial cells (mTEC)	Medullary thymic epithelial cells are the major subset of antigen presenting cell in the thymus. mTEC play a key role in the negative selection of thymocytes, which ensures that thymocyte expressing TCR against self-antigens are removed.
TGF-β	A key cytokine involved in promoting Treg development, by promoting the expression of the transcription factor FoxP3. FoxP3 is a master regulator of Treg differentiation and function.
IL-10	A key cytokine produced by regulatory T cell involved in immune suppression. IL-10 can also be produced by other cell types to promote Treg differentiation.
Pathogen associated molecular pattern (PAMP)	Pathogen associated molecular pattern are conserved microbial motifs that are recognized by pattern recognition receptors such as toll-like receptors (TLR). A common PAMP is lipopolysaccharide (LPS), which is expressed by gram-negative bacteria.
Extracellular vesicles (EV)	Extracellular vesicles are nano-sized particles released by all cell types via the budding of the plasma membrane. They can cargo nucleic acid, proteins and metabolites. Bacterial-derived EV has been shown to interact with host cells, activating TLR to promote Treg differentiation.
Short-chain fatty acids (SCFA)	Short-chain fatty acids are the major metabolite produced by gut bacteria during the fermentation of dietary fiber. SCFA are sensed by the host, and they can directly promote Treg differentiation.

## TREG Development

### Thymic Treg Differentiation

#### Overview

The thymus is a specialized primary lymphoid organ and is the site of thymopoiesis, a process by which mature and functional T cells are generated. The earliest T cell progenitors are bone marrow-derived hemopoietic stem cells (HSC) that migrate to the thymus through their expression of chemokine receptors CCR9 and CCR7, as well as P-selectin ligand (11). The first step of thymopoiesis is characterized by the generation of the early T lineage progenitors, the earliest T cell lineage-committed HSCs. These progenitors differentiate into CD4^−^CD8^−^ double-negative and then to CD4^+^CD8^+^ double-positive thymocytes, which undergo several developmental checkpoints called positive and negative selection, which occur in different anatomic parts of the thymus (7). These processes ensure the strict selection of thymocytes with functional T-cell receptors (TCR). These TCR bind effectively self-major histocompatibility complex (MHC) but are hyporesponsive to self-antigens. Positive selection occurs within the thymic cortex, where CD4^+^CD8^+^ double-positive thymocytes interacting effectively with self-MHC molecules expressed by cortical thymic epithelial cells survive. These cells further differentiate into single positive CD4^+^CD8^−^ or CD8^+^CD4^−^ thymocytes. These single positive thymocytes then migrate to the medulla, where they undergo the negative selection, a process eliminating autoreactive thymocytes with high affinity to self-antigens. Self-antigens are presented by various antigen-presenting cells (APCs), particularly medullary thymic epithelial cells (mTECs). Strong TCR signaling to self-antigens leads to clonal deletion by apoptosis, while a small percentage survive to become Tregs. The mechanisms behind the commitment of autoreactive CD4^+^ T cell toward Tregs differentiation rather than clonal deletion is not fully understood but likely involves multiple factors, including affinity of TCR binding to self-antigen/MHCII complex, duration of TCR signaling, and the phenotype of the APCs presenting the antigens (discussed later) (7). Regardless, TCR interactions with self-antigen appear to be the dominant signal for tTreg induction. It was elegantly demonstrated by utilizing T cells with transgenic TCR of varying affinities to OVA peptide that mice with higher-affinity TCR-bearing T cells have a larger tTreg pool (12). Other factors, such as costimulation and cytokines, support their development and maturation. Engagement of the co-stimulatory molecule CD28 to CD80/CD86 ligand expressed on APCs augmented the TCR signaling pathway to promote tTreg differentiation (13) and survival (14). The presence of CD28 on T cells was correlated with increased diversity of the TCR repertoire of the tTreg pool, possibly by promoting tTreg differentiation from thymocytes expressing infrequent TCRs (14). The pro-survival cytokine IL-2, which signals through CD25 in an autocrine manner (15), was required for early tTreg survival and expansion and later maturation (16). A deficiency of IL-2 could be compensated by other pro-survival cytokines such as IL-7 and/or IL-15 expressed by mTECs (17, 18).

#### Antigen-Presenting Cells in the Thymus and Their Role in tTreg Induction

mTECs are the main cell subset involved in antigen presentation in the thymus, a process regulated by the transcription factor autoimmune regulator (AIRE). AIRE, along with Fez family zinc finger protein 2 (FEZF2), regulates the expression of tissue-specific antigens (TSAs) for presentation to thymocytes. Together, they coordinate ~60% of the TSAs expressed by mTECs (19). FEZF2 and AIRE control the expression of a mostly distinct set of TSA genes, although almost 40% of TSAs among mTECs were regulated through unknown mechanisms independent of AIRE and FEZF2 (19). This suggests that other transcription factors or potential extrathymic factors regulate the tTreg repertoire.

Apart from mTECs, other APCs can contribute to tTreg differentiation by presenting a distinct repertoire of antigens (20), suggesting that mTEC and other APC have a complementary role in establishing optimal tolerance (21, 22). These APCs include dendritic cells (DCs), plasmacytoid DCs (pDCs), and B cells, which have varying abilities to promote tTreg generation, as reviewed previously (23). Intrathymic-derived CD8α^+^ conventional type I DCs present thymic-derived antigens acquired by membrane transfer from mTEC (24) but are dispensable for tTreg induction (22). On the other hand, extrathymically-derived SIRPα^+^ conventional type 2 DC, which can capture peripheral antigens for presentation to thymocyte, were critical for tTreg development (25). Other peripheral APCs can also capture peripheral antigens and egress to the thymus, including PDCA-1^+^ pDC (25) and gut-derived CX3CR1^+^ DCs (26). While gut-derived CX3CR1^+^ DCs have been shown to present microbiota-derived antigens and promote the generation and expansion of microbiota-specific thymic T cells, they did not appear to affect tTreg development (27). Similarly, thymic pDC are weak Treg inducers *in vitro* (26) and are unlikely to play a role *in vivo* (26). Altogether, mTECs are the main tTreg inducers, yet more research is needed to confirm the role of other APCs in tTreg development.

#### Dynamic of tTreg Development at Different Stages of Life

The biology of T cell development fluctuates throughout life. For example, early fetal early thymic progenitors (ETP) are biased toward T cell differentiation (E12–E15), while later waves of ETP (after E16) have myeloid differentiation potential that more closely represented postnatal ETP (28). CD4^+^ T cells generated in the fetal thymus also produce more cytokines and are skewed toward Th2 differentiation, while adult thymic-derived CD4^+^ T cells produce fewer cytokines and are skewed toward Th1 cytokine production (29).

Differences in the aging thymic environment also affect tTreg development, with thymic progenitors having a reduced propensity toward Treg differentiation with increasing age (30). This is likely an evolutionary mechanism to maintain a tolerogenic environment during pregnancy and exposure to environmental antigens during early life. The neonatal tTreg pool is also characterized by a greater TCR repertoire diversity and increased expression of FoxP3 and suppressive molecules, such as CTLA-4, compared to adult Tregs (31, 32). The more diverse neonatal tTreg TCR repertoire likely results from the ability of perinatal mTECs to present a more extensive repertoire of TSAs (33). These age-dependent differences suggest a developmental window for the selection of tTregs with specific clonotypes. One example is the development of Padi4-specific tTregs, which was restricted to the neonatal period (34). The inability of the adult thymus to generate Padi4-specific tTregs appears to relate to the presentation of the Padi4 peptide by non-mTEC APCs instead of mTECs, which promotes instead clonal deletion. mTECs are the major tTreg inducer in the neonatal thymus, as other APCs (such as pDC and conventional DC) do not efficiently seed the thymus until later in life (35). Other cells maintain the same function throughout life, such as colonic-derived pDCs. These pDCs are regulated by the neonatal gut microbiota and migrate to the thymus to support PLZF^+^ innate-like T cell differentiation, with the effect persisting into adulthood (36). These PLZF^+^ innate-like T cells contribute to the differentiation of thymic Treg with an activated/memory-like phenotype (37).

### Peripheral Treg Differentiation

While tTreg and pTreg are characterized by their expression of FoxP3 and their immunosuppressive functions, they differ in their ontogeny. pTreg can arise from naïve CD4^+^ T cells dependently or independently of peripheral APC. Contrary to tTreg, pTreg differentiation mainly relies on environmental cues, particularly the cytokine tumor growth factor-β (TGF-β) that promotes the upregulation of FoxP3 (38). The commitment to the Treg lineage also depends to a lesser extent on TCR signaling and engagement of co-stimulatory molecules such as CTLA-4 (39). TGF-β promotes the differentiation of Tregs via phosphorylation of Smad3, which then translocates to the nucleus and acts as a transcription factor regulating the expression of *Foxp3* (40). Non-cytokine factors can also direct FoxP3 expression and promote *de novo* pTreg differentiation via epigenetic regulation of the *Foxp3* locus. FoxP3 expression is controlled by three major regulatory regions within the FoxP3 locus: the conserved non-coding DNA sequence (CNS) elements CNS1, CNS2, and CNS3. Demethylation of the CNS regions is observed in both tTregs and pTregs, with CNS1 appearing to be critical for pTreg but not tTreg differentiation (41, 42). Similarly, acetylation of histone H3 both at the promoter and CNS regions of the *Foxp3* locus promoted pTreg differentiation (43).

Numerous immune and non-immune cells can provide the environmental cues necessary for pTreg differentiation. Among the immune cells, CD103^+^ DCs, macrophages, regulatory B cells (Bregs), and Treg support pTreg differentiation through their release of TGF-β. Antigen-specific pTregs are induced by TGF-β and retinoic acid produced by gut mucosal CD103^+^ DC and lung resident macrophages, protecting from food allergies, asthmatic lung inflammation, and airway hyperreactivity, respectively (44). Bregs produce both TGF-β and IL-10 to induce pTregs (45), ensuring allograft tolerance (46). Activated Tregs can also promote the differentiation of Tregs from naïve CD4^+^ T cells through their surface expression of TGF-β, which activates contact-dependent TGF-β signaling (47). CD103^+^ DC, gut epithelial and stromal cells are also a source of TGF-β and retinoic acid, supporting pTreg differentiation to maintain oral tolerance (48–50).

## Impact of Diet on TREG Development

**Figure 1:**
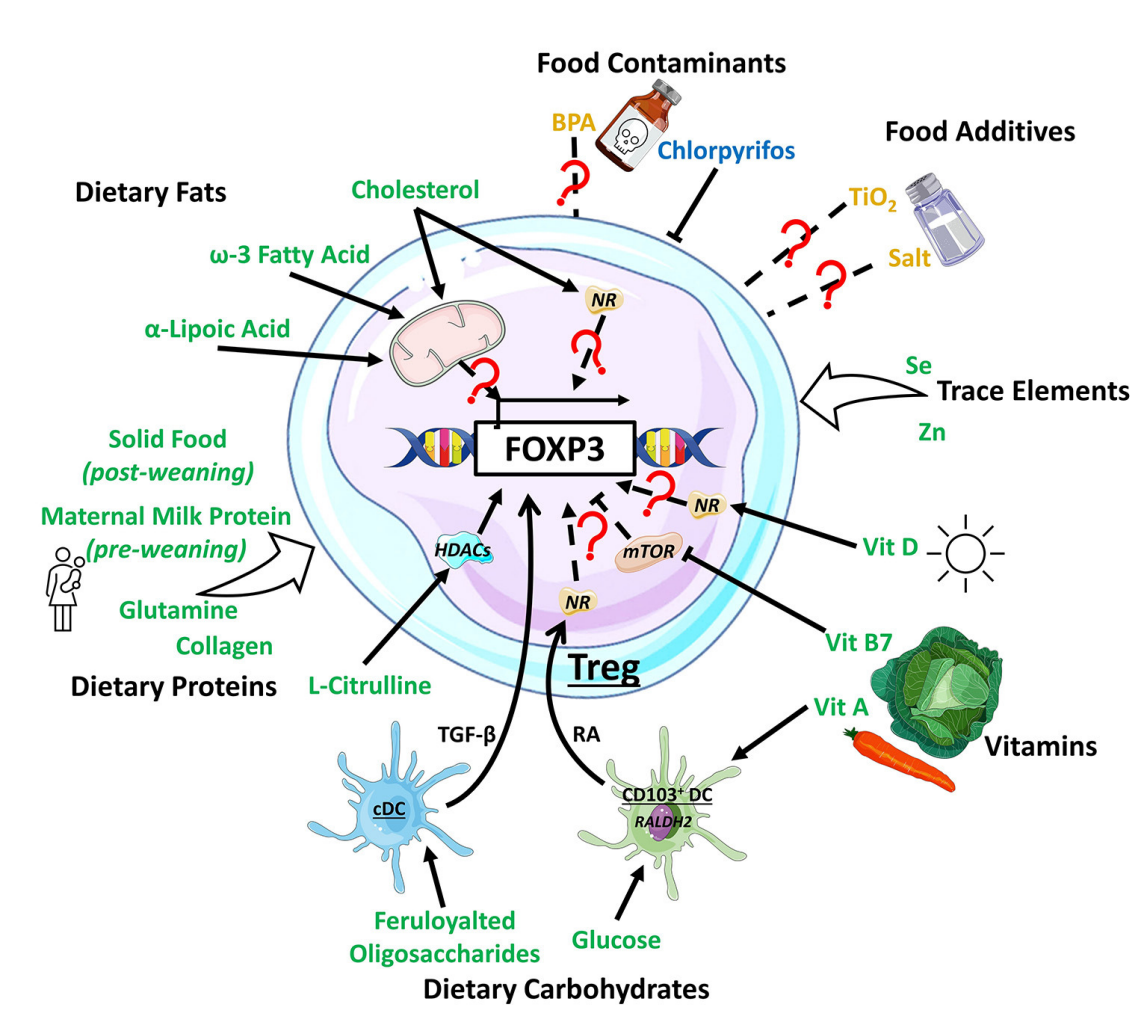
Impact of dietary components on Treg development. Dietary components have varying effect on Treg development, with dietary fats inducing Treg by modulating metabolic activity or through the activation of nuclear receptors (NRs) such as PPARδ. Dietary protein generally support Treg development, with L-citrulline enhancing the histone deacetylase (HDAC) Sirt1 to promote Treg development. Dietary carbohydrates, including feruloylated oligosaccharides and glucose act on conventional dendritic cell (cDC) and CD103^+^ DC, boosting their production TGF-β and retinoic acid (RA), respectively, to promote Treg. Vitamin (Vit) A is a substrate for RA production required for Treg induction while vitamin B7 and D finetune mTOR-related metabolic signals or bind to NRs to support Treg induction. Dietary trace elements like selenium (Se) and zinc (Zn) are supportive toward Treg development with unknown mechanisms. The food additives TiO_2_ and salt, and the contaminant biphenol A (BPA) exhibit mixed/complex regulatory patterns toward Treg development while pesticides like chlorpyrifos suppressed Treg development. Dietary components promoting Treg development are colored in green, the ones impeding in blue, and the ones with mixed effect in yellow.

Diet is a major environmental factor influencing health. A balanced diet consists of 45–65% carbohydrates, 20–35% fat, and 15–25% protein by energy content and sufficient intakes of micronutrients, such as minerals and vitamins. While the role of diet on metabolic health is well-established, an increasing number of studies reveal its impact on the immune system, particularly on Treg differentiation and function. Diet may directly or indirectly affect Treg through changes in the gut microbiota (51). A summary of how different dietary components promote the differentiation of Treg is presented in Figure 1.

### Influence of Diet on tTreg Ontogeny

The role of diet in tTreg induction is not well-established. Nonetheless, two factors related to diet promote tTregs: short-chain fatty acids (SCFA), a by-product of the fermentation of complex carbohydrates by gut bacteria, and dietary cholesterol. The role of SCFA on tTreg ontogeny will be discussed in the gut microbiota section below. Cholesterol, derived from animal products, is prevalent in the Western diet, and supplementation of a standard murine chow with 0.15% cholesterol enhanced TCR responsiveness in CD4^+^ T cells and increased Treg frequency and suppressive capacity in both central and peripheral compartments (52). The mechanisms behind these effects remain elusive.

On the other hand, dietary salt has been shown to reduce the expansion of thymic tTregs and decrease their suppressive function when mice were fed on a high-salt diet. Dietary salt changed extracellular osmotic pressure, altering cell tonicity by activating the tonicity-responsive enhancer-binding protein and p38 signaling pathway. These changes resulted in the upregulation of serum/glucocorticoid-regulated kinase 1, promoting IFNγ and RORγt expression. As a result, tTregs were repolarized toward Th1 and Th17 lineage. Serum/glucocorticoid-regulated kinase 1 also phosphorylates and inhibits FOXO1 and FOXO3, decreasing FoxP3 expression and thus tTreg differentiation (53). Interestingly, these effects are specific to murine tTreg and not pTreg, with further investigation needed to clarify whether these effects apply to humans.

### Influence of Diet on pTreg Ontogeny

#### Macronutrients and pTreg

##### Role of Dietary Fats

Dietary fats, such as cholesterol and omega-3 fatty acids, and fat metabolic intermediates, such as α-lipoic acid, increase pTreg populations. The impact of fatty acids and cholesterol on pTreg induction is mostly via metabolic effects, with fatty acid oxidation promoting Treg differentiation. Oral administration of α-lipoic acid in obese mice restored visceral Treg numbers by enhancing naïve T cell oxidative capacity and fatty acid oxidation (54). Similar results were observed in humans, in obese women who received α-lipoic acid supplementation (54). Likewise, in mice treated on a diet containing 0.25% cholesterol and 15% cocoa butter, the accumulation of cholesterol in Treg increased fatty acid oxidation, leading to an increased splenic Treg population (55). Cholesterol also promotes Treg development by activating mammalian target of rapamycin (mTOR) complex 2 and PPARδ signaling (55). Fatty acids may also indirectly induce pTreg differentiation with for example ω-3 docosahexaenoic acid induced production of lipoxin A4 by neutrophils, which increases pTregs (56, 57).

The type and the amount of dietary fat consumed can modulate the pTreg compartment. pTreg are decreased in tissues such as the spleen and white adipose tissues in both high-fat diet (HFD) induced obesity (58) and non-alcoholic steatohepatitis (59) animal models, as well as in a humanized mouse model (60). This was paralleled with low-grade inflammation and metabolic alteration. Similarly, HFD-induced myocardial fibrosis and atherosclerosis in mice were characterized by decreased Tregs in the heart (61) and aorta (62). Dietary fat may also affect pTreg function, as mice fed an HFD containing 35% hydrogenated vegetable oil had decreased IL-10 (60). Whether these Treg number and activity changes are directly due to HFD or are secondary to metabolic alteration is unclear. Changes in the oxidative environment linked to metabolic alteration may directly contribute to decreased Treg. In an HFD-induced non-alcoholic steatohepatitis model, the rise of hepatic oxidative stress and reactive oxygen species triggered Treg apoptosis, increasing liver inflammation (63).

Conversely, isocaloric ketogenic diets consisting of high fat (70–80%) and low carbohydrate (5–10%) content increase naïve T cell fatty acid oxidation, promoting Treg differentiation and IL-10 production (64). To our knowledge, only one study has investigated the impact of a ketogenic diet over 3 weeks on Treg induction in humans (64). Data in mice are inconsistent depending on the model with either an increase in Tregs in a carcinomatous peritonitis model (65) or a decrease in a diabetic obese mouse (66). This difference may be due to the altered metabolic status of the mice.

##### Role of Dietary Proteins

Dietary proteins are the primary source of food antigens, a key component of pTreg development. Before weaning, the maternal breastmilk proteins constitute the majority of luminal antigens contributing to colonic pTregs generation. Colonic Treg from such origin plays an important role in tolerance maintenance and allergy suppression by dampening the Th2 responses (67, 68). Unlike Tregs in the colon, Tregs in the small intestine lamina propria are induced during weaning when exposure to solid food commences. This population is sparse before weaning, implying breastmilk has a limited contribution to their development. There is, however, a significant boost in their induction upon weaning with a regular chow diet, both in germ-free (GF) or specific-pathogen-free (SPF) mice. Conversely, weaning with an antigen-free diet (deficient in dietary protein antigens) fails to replenish small intestine pTreg. Similar observations have been reported in zebrafish, where pre-exposure to food antigens promoted an intestinal Treg phenotype while lack of prior food antigen exposure did not (69).

Studies centered on the impact of dietary protein on pTreg have primarily focused on specific proteins or amino acids. Cow milk proteins have been implicated in pTreg development, although changes are dependent on the type of protein. Four generations of mice were fed from birth with a nutritionally balanced milk-based diet containing either A1 or A2 beta-casein components. While there were no changes in conventional FoxP3^+^CD25^+^ Tregs, the A2-fed cohort had increased non-conventional FoxP3^+^CD25^−^ Tregs in the spleen (70). Regarding the impact of specific amino acids on Treg development, glutamine supplementation has been the most investigated. A diet in which 25% of casein was replaced by glutamine increased circulating pTregs in both sepsis (71) and DSS-induced colitis mouse model (72), though the mechanism was unresolved. Other amino acids such as collagen and L-citrulline also increased pTreg differentiation *in vitro* and *in vivo*, respectively (73). The effect of L-citrulline on naïve T cells was linked to the upregulation of the deacetylase Sirtuin 1 (Sirt1) and downregulation of Smad7, an inhibitor of TGF-β signaling. These changes enhanced *Foxp3* transcription and IL-10 production, thus Treg differentiation (73).

##### Role of Dietary Carbohydrates

Carbohydrates such as feruloylated oligosaccharides and glucose can increase pTreg differentiation (74, 75). Oral administration of 200 or 400 mg/kg/day of feruloylated oligosaccharides increased colonic Tregs in a DSS-induced colitis mouse model, likely through the increased production of TGF-β from DCs (75). The *in vitro* addition of glucose to isolated small intestine-derived DCs increased RALDH2 activity, leading to higher retinoic acid production and enhanced ability to promote Treg differentiation (74).

#### Micronutrients and pTreg

##### Zinc and Selenium

Micronutrients such as zinc and selenium modulate pTreg generation. A zinc-deficient diet significantly decreased splenic pTreg without altering their suppressive function in a mouse model of dermatitis (76). Conversely, a diet enriched in zinc increased pTreg numbers in weaned piglets and adult pigs (77, 78). The mechanism(s) behind zinc's effect on Treg remains elusive, as is whether this effect applies to human Treg. Selenium supplementation in drinking water protected mice from autoimmune thyroiditis (79) and DSS-induced colitis (80) by inducing splenic and colonic lamina propria pTreg, respectively. During DSS-induced colitis, selenium also increased IL-10 production in CD4^+^ T cells, reducing colonic inflammation. The role of selenium in human Treg biology is under-investigated. Only one study demonstrates that daily selenium supplementation (200 μg/day) for 3 months had no impact on a subset of non-Hodgkin lymphoma patients (81).

##### Vitamins

Deficiency in vitamins D, B7, and A, has been shown to reduce both the proportion and functionality of pTreg. Vitamin D deficiency reduced splenic Tregs in healthy mice (82) and decreased Treg in sino-nasal tissue during chronic rhinosinusitis (83). Similarly, vitamin B7 deficiency reduced the expression of FoxP3 in murine inguinal lymph nodes and inhibited Treg differentiation *in vitro*. These effects involved mTOR activation, which inhibited FoxP3 expression in CD4^+^ T cells while promoting IFNγ and IL-17 (84). Vitamin A is a crucial factor promoting Treg development via its conversion into retinoic acid by the enzyme RALDH2 expressed in CD103^+^ DC. The absence of vitamin A impairs Treg generation and suppressive function (6).

##### Food Additives

Food additives are prevalent in modern diets and added to processed food for various reasons, such as increasing shelf-life and improving texture. The whitening agent titanium dioxide (TiO_2_) is one of the most common food additives (85, 86), and its reported effects on pTreg appear inconsistent. While water supplementation with TiO_2_ (10 mg/kg body weight/day) for 7 days reduced Tregs in Peyer's patches in rats (87), the addition of TiO_2_ into food had no impact (88). In mice, the addition of TiO_2_ to drinking water for 3–4 weeks at either a physiological (10 mg/kg body weight) or high-dose (50 mg/kg/body weight) had no impact on Treg (85). Emulsifiers are used to improve food texture and have been shown to exacerbate obesity and colitis in mice via effects on the gut microbiota (89). Despite the reported pro-inflammatory effect of emulsifiers carboxymethylcellulose or polysorbate-80, their impact on Treg is unknown.

Salt has been used as a preservative for millennia, and its impact on pTreg varies in humans and mice and depends on individuals' health status. In a pilot study of 5 healthy men, 2 week-treatment on a high-salt diet did not alter pTreg numbers (90), while in healthy mice, a high-salt diet reduced bone marrow Treg and impaired their function, as they had decreased ability to produce IL-10 (91). Dietary salt consumption was negatively correlated with circulatory CD69^+^ Treg and NKG2D^+^ Treg in rheumatoid arthritis and systemic lupus erythematosus, respectively (92). Decreased salt consumption restored the circulating pTreg pool in these patients (93). On the other hand, high-salt feeding had a negligible effect on pTreg in the spleen, draining lymph nodes and mesenteric lymph nodes in a mouse model of multiple sclerosis (94). Sodium benzoate, another commonly used food preservative, has been shown to induce pTreg by increasing TGF-β via STAT6 dependent mechanisms, protecting mice from experimental autoimmune encephalomyelitis (95).

##### Food Contaminants

Modern agricultural practices expose food to unwanted contaminants such as pesticides. In mice, intake of 7 mg/kg chlorpyrifos, a widely used organophosphate pesticide, decreased the circulatory Treg pool and suppressed Treg-related gene expression in the spleen (96). While pesticide exposure has extensive links to specific diseases such as cancer (97), their impact on the immune system is understudied. Plastic food packaging is also a significant source of food contamination, particularly packaging containing the synthetic organic chemical bisphenol A (BPA). However, the role of BPA on Treg is inconclusive, with one study finding that mouse exposure to BPA at different developmental stages decreased splenic Tregs in males (98, 99) and another study finding the opposite (100). While not strictly a contaminant, plant cells like any cells, can also produce extracellular vesicles (EV) either under basal conditions or in response to threats. These plant-derived particles have been shown to modulate mammalian cell phenotypes, including immune cells. For instance, plant-derived exosome-like nanoparticles derived from ginger and carrot induced IL-10 production in RAW264.7 macrophage cell line (101) but whether this effect applies to T cells is unknown. Similarly, whether plant-derived EV likely present in food products are immunomodulatory *in vivo* and affect Treg development remain elusive. Plant-derived EV can modulate bacterial growth (102), and thus, as a result, may alter the gut microbiota composition. As discussed below, changes in gut microbiota may lead to Treg development.

#### Caloric Restriction

Caloric restriction (CR) has been shown to promote pTreg, protecting mice from experimental autoimmune encephalomyelitis (103, 104) and ischemic stroke (105). By limiting T cell energy availability, CR raises the intracellular ratio of adenosine monophosphate (AMP): adenosine triphosphate. This activates AMP-activated protein kinase, which inhibits mTOR promoting Treg differentiation. CR downregulate acetyl-coenzyme A carboxylase 1, decreasing *de novo* fatty acid synthesis and biasing naïve T cell differentiation toward Treg rather than Th17 (105). CR also elevates ketone bodies, but this does not contribute to pTreg differentiation, as murine supplementation with ketone ester (a ketone body raising agent) did not affect splenic and intestinal Tregs (106).

## Gut Microbiota and TREG Development

**Figure 2:**
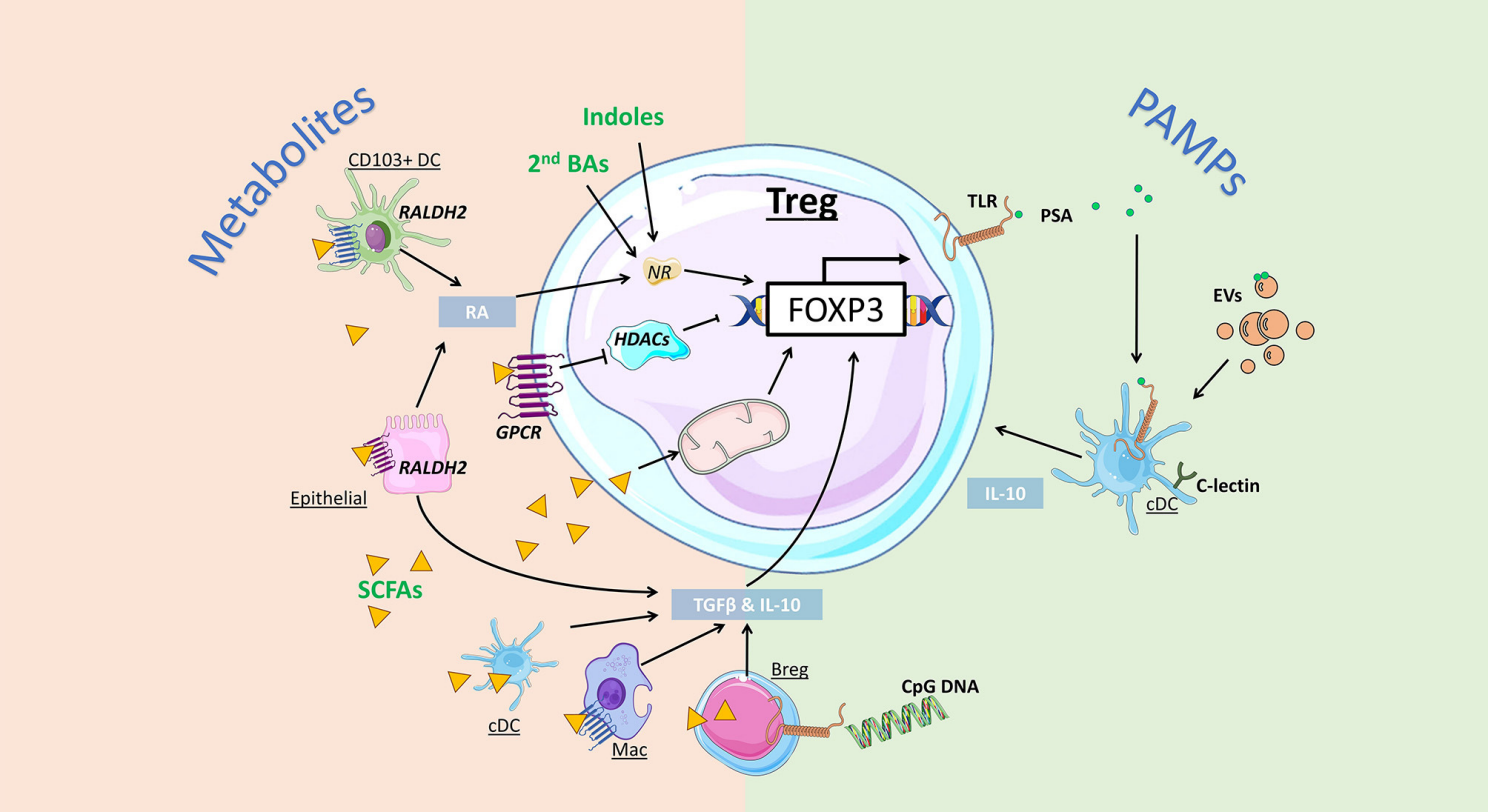
Impact of gut bacteria on regulatory T cell development and function. Microbiota-derived metabolites can regulate Treg development and function via distinct mechanisms. Indoles and secondary bile acids (BAs) can bind to nuclear receptors (NRs) to promote *Foxp3* expression and Treg induction. Short-chain fatty acids (SCFAs) can activate G protein-coupled receptors (GPCRs) to promote Treg directly via inhibition of histone deacetylase (HDAC) activity, or indirectly, by enhancing retinal dehydrogenases (RALDH) and retinoic acid (RA) production by CD103^+^ DCs and epithelial cells. Alternatively, SCFA are directly taken up by cells, including regulatory B cells (Bregs) and conventional DCs (cDCs) to promote expression of Treg-inducing cytokines TGF-β and IL-10. SCFA uptake by T cells can also directly promote Treg induction by increasing mitochondrial activity. Bacterial pathogen associated molecular patterns (PAMPs) can also promote Treg via activation of toll-like receptors (TLR). *Bacteroides fragilis-*derived polysaccharide A (PSA) promote Treg via TLR signalling on DCs, promoting IL-10 production, as well as Treg-intrinsic TLR2 signals. Extracellular vesicles (EVs) are PAMPs that can bind to TLR and C-lectin expressed by gut DCs and epithelial cells, triggering the release of Treg-inducing cytokines IL-10 and TGF-β.

Bacteria, viruses, archaea, and fungi colonize the human gastrointestinal tract from birth. Collectively, this community is known as the gut microbiota and has diverse effects on the host. The gut microbiota can influence host metabolic, physiological, and immunological functions and mediate these effects via direct (i.e., membrane components) or indirect (i.e., production of metabolites) mechanisms (107). The overall impact of bacteria-derived metabolites or antigenic components is summarized in Figure 2.

The intestinal compartment is particularly enriched in Tregs, representing approximately 30% of colonic lamina propria CD4^+^ T cells (108). Most studies indicate that colonic Tregs are generated *de novo* through contact with the microbiota (109, 110), with only one study reporting colonic Tregs are thymic-derived (111). Indeed, compared to SPF mice, GF mice have reduced numbers of colonic Tregs (109). Antigenic stimuli from the gut microbiota generate and activate RORγt^+^ Tregs, a critical subset of colonic Tregs, which are integral to intestinal homeostasis (110). RORγt^+^ Tregs, which comprise 65% of colonic Tregs, require microbial antigens and are absent in GF conditions (112, 113). RORγt^+^ Tregs expand during the weaning period when there is an influx of food and microbial antigens. These cells suppress both Th1 and Th2 responses and maintain homeostasis with commensal microbiota (112). A variety of microbes can induce colonic RORγt^+^ Tregs in GF mice, including bacteria from *Clostridia* (109)*, Bacteroides* (113), and to a lesser extent, *Segmented Filamentous Bacteria* (113). As discussed later, other bacterial strains, listed in Table 2, have been reported to influence Treg development through various mechanisms.

**Table 2:** Impact of bacteria on Treg phenotype.

**Bacteria**	**Model**	**Impact on Tregs**	**Mechanism of Treg induction**	**Effect on immune system/ disease**	**References**
*Helicobacter pylori* (likely mediated by CagA^+^ strains)		↑ Splenic Tregs	DC-derived TGF-β	Presence in microbiota associated with decreased asthma, allergic disease, Inflammatory bowel disease	(114–118)
		↑ FoxP3^+^ Treg induction *in vitro*	Bone marrow-derived DC secretion of IL-10 via TLR2	↑ gastritis and inflammation during *H. pylori* infection in *Tlr*2^−/−^ mice	
		↑ CD25^+^ T regulatory cell	Breg secretion of IL-10 via TLR2 activation	↓ Gastric pathology *in vivo*	
		↑ Pulmonary Tregs	DCs	↓ airway inflammation in neonatal and adult mice	
		↓ Mesenteric lymph node Tregs in *Il*−18^−/−^ mice	Induction of IL-18 producing DCs	Transfer of CD25^+^ cells from *Il*18^−/−^ or *Il*18*r*^−/−^ mice lacked suppressive activity during airway allergic disease model	
7 strain mix: *Lactobacillus acidophilus* CBT LA1*, Lactobacillus rhamnosus* CBT LR5*, Lactobacillus plantarum CBT LP3, Bifidobacterium bifidum CBT BF3, Bifidobacterium breve CBT BR3, Lactococcus lactis CBT SL6, Streptococcus thermophilus CBT ST3*	SPF	↑ CD25^−^FoxP3^+^ Tregs in MLNs, Foxp3 mRNA in skin		↓ serum IgE, Th2 cytokines, dermatitis symptoms	(119)
5 strain mix (IRT5): *Lactobacillus acidophilus, Lactobacillus casei, Lactobacillus reuteri, Bifidobacterium bifidium, Streptococcus thermophilus*	SPF	↑ CD4^+^Foxp3^+^ Tregs in MLNs of healthy mice	Tolerogenic CD103c^−^ CD11c^+^ DCs expressing IL-10, TGF-β, Cox-2, Indoleamine-pyrrole 2,3-dioxygenase	↓ disease scores in Inflammatory bowel disease, atopic dermatitis, rheumatoid arthritis models, ↓ Th1, Th2, Th17 responses in T and B cells *in vitro*	(120)
*Lactobacillus paracasei KBL382*	SPF	↑ CD25^+^ Foxp3 Tregs in MLNs	Altered cecal microbiota profile, ↑ SCFAs and lactate, succinate, and fumarate	↓ serum IgE, atopic dermatitis symptoms	(121)
*Bacteroides thetaiotaomicron VPI 5482 and 29148*	SPF	↑ CD4^+^Foxp3^+^ cells, CD4^+^ICOS^+^ T cells, CD4^+^ICOS^+^ Foxp3^+^ regulatory T cells, and IL-10-expressing CD4^+^Foxp3^+^ cells in spleen, MLN, and cervical lymph nodes	Possibly ↑ SCFA	↓ OVA-specific IgE airway inflammation	(122, 123)
		RORγt+ Treg homeostasis in the gut	Bile acid	Protection from colitis	
8 defined species of Altered Schaedler Flora	GF	↑ Helios^−^ Treg, activated CD103^+^ Treg, *de novo* generation of colonic Tregs of C57BL/6, BALB/c, Swiss, Webster, NMRI mice	TLR signalling through MyD88 and Ticam-1, independent of IL-10R signalling	Select monocolonisation unable to recapitulate effects of full Altered Schaedler Flora	(124)
*Akkermansia muciniphila* BAA-835, 139	SPF	↑ FoxP3^+^ Tregs in MLNs ↑MOG-specific FoxP3^+^ Tregs, total splenic Tregs, FoxP3^+^ Tregs in visceral adipose tissue	↑ cecal SCFA concentration ↑TGF-β and ↓IL-6, IL-1β from MLN DCs ↑ ileal goblet cells which likely encourages tolerogenic DCs, ↓ IL-6, IL-1β in visceral adipose tissue	Normalised gut microbiota, ↑ recovery from DSS-colitis ↓ disease score in MS model improved metabolic profile in HFD-induced obesity	(125, 126)
*Bifidobacterium infantis* 35624	SPF	↑ CD25^+^ Tregs in Peyer's Patch, CD25^+^ FoxP3^+^ splenic Tregs	Possibly ↓CD80 expression by DCs in Peyer's Patches and spleen ↑	↓ NF-κB activity, protection from *S. typhimurium*	(127, 128)
	Human	↑ FoxP3^+^ expression in peripheral blood CD4^+^ cells after oral feeding human volunteers for 8 weeks	IL-10 secretion, RALDH2, Indoleamine-pyrrole 2,3-dioxygenase expression in DC subsets *in vitro* via PRR activation		
*Bifidobacterium longum* AH 1206	GF, SPF	↑ FoxP3^+^ Tregs in Peyer's Patch and spleen of infant, adult and GF models	Possibly altered genes of retinoic acid metabolism in Peyer's Patches	↓ serum OVA-IgE, airway inflammation	(129)
*Bacteroides fragilis* NCTC934	SPF	↑ RORγt+ colonic Tregs	Bile acid metabolism	Bile acid supplementation protected from colitis	(130–132)
*Bacteroides fragilis* 9343	GF	↑ IL-10+FoxP3+ colonic Tregs; enhanced CD4+ conversion to FoxP3+ *in vitro*	PSA induces tolerogenic DCs via TLR2	PSA protects from colitis; protects from MS model	
*Lactobacillus fermentum* KBL374, KBL375	SPF	↑CD25^+^Foxp3^+^Tregs in MLNs	↑ IL-10 colonic expression, *Akkermansia* expansion, altered cytokine expression	Protection from DSS colitis, normalised microbiota composition	(133–135)
		↑ CD25^+^Foxp3^+^ Tregs in MLN, Foxp3^+^ expression in skin	↑ IL-10 in skin, SCFA concentration in cecum	↓ serum IgE, atopic dermatitis, altered cecal microbiota	

Although many studies examine singular mechanisms for Treg induction, it is unlikely that bacteria conform to any one mode of action. For example, 46 strains from the genus *Clostridia*, derived from Clusters IV and XIVa, induced colonic Tregs in GF mice (109). This was attributed to increased secretion of TGF-β and indoleamine 2,3-dioxygenase (a catabolic enzyme for tryptophan) by colonic intestinal epithelial cells, which induced FoxP3^+^ Tregs independently of pattern recognition receptor (PRR) signaling. Treg levels were preserved at least 4 months post-induction, and this increased Treg phenotype was vertically and horizontally transmissible to other GF mice along with microbiota composition, indicating that the *Clostridia* clades could stably induce Tregs. The mechanism behind *Clostridia* Treg-induction appears multifactorial, dependent on metabolites (SCFA and indole) (136), bacterial antigens, and Treg-promoting cytokines, particularly TGF-β1 in both mouse and human epithelial cell lines (136). The authors also identified *Clostridia*-specific T cells, suggesting specific antigens contributed to Treg induction (136). Monocolonization of GF mice with single strains of *Clostridia*, however, was unable to fully recapitulate the Treg-inducing capability of the 17-strain mix, indicating the microbiota cooperatively promoted Tregs through independent mechanisms. Members of *Clostridia* predominantly ferment dietary fiber or indigestible carbohydrates (particularly polysaccharides) as an energy source; however, some *Clostridium* species preferentially utilize amino acids/protein as a nutrient source [reviewed in (137)]. Supplementation with different fibers promotes different *Clostridia* clusters, in distinct GI tract sites, with piglets fed on a diet supplemented with either alfalfa or cellulose for 3 weeks had increased cluster XIVa in the distal and proximal colon, respectively (138). Overall, bacterial species induce Tregs through multiple mechanisms, which can be dramatically affected by diet.

## Gut Bacterial Metabolites and PTREG Development

### Metabolites

#### Short-Chain Fatty Acids

SCFAs are by-products of bacterial fermentation of dietary fiber. The three predominant SCFAs, acetate, butyrate, and propionate, ameliorate inflammatory diseases by regulating Treg function and frequency (139–141). SCFAs possess intrinsic histone deacetylase (HDAC) inhibitory properties and directly promote *Foxp3* gene expression. They induce pTreg differentiation through the acetylation of histones H3 and H4 within the *Foxp3* locus (42, 43, 139–142). Deficiency in GPR43, a receptor for acetate and propionate, counteracted the effect of propionate on the acetylation of histone H3K9 (139). As a result, propionate treatment in GPR43 knockout mice did not increase Treg (139). However, other studies have reported no defect in Treg development in both *Gpr*43^−/−^ and *Gpr*41^−/−^ mice (142). SCFAs can also promote Treg differentiation via HDAC-independent mechanisms by altering Treg metabolism. Propionate treatment in multiple sclerosis patients enhanced Treg oxygen consumption rate, altered their mitochondrial morphology, and their improved suppressive functions. These patients had increased proportion of circulating Tregs, which ameliorated disease progression (143).

SCFAs can indirectly upregulate Tregs through their actions on other immune cells. Activation of GPR109A by butyrate in DCs and macrophages increased *Aldh1a1* and *Il10* expression, improving their ability to induce Treg differentiation and function (144). Moreover, acetate and butyrate treatment in a murine food allergy model increased the number and activity of CD103^+^ DCs, which enhanced the generation of antigen-specific Tregs and protected against severe anaphylaxis (6). Bregs also promote Tregs via their secretion of TGF-β (145) and IL-10 (146). Acetate can directly induce Bregs via stimulation of the tricarboxylic acid cycle and post-translational acetylation (147, 148). Current literature, however, is fraught with inconsistencies; Daïen et al. (148) found acetate promoted Bregs while butyrate was inhibitory, whereas Zou et al. (147) observed the opposite.

Interestingly, acetate could not induce Tregs *in vitro* when combined with TGF-β (42) but did upregulate IL-10 production and FoxP3 expression under Th1/Th17-polarizing conditions (142). In mice, acetate upregulated *Foxp3* only after induction of allergic airway disease (140). T cell transfer from non-obese diabetic mice fed an acetate-enriched diet could not protect their peers from induction of disease, unlike T cells transferred from their counterparts fed a butyrate-enriched diet (141). Similarly, propionate-mediated amelioration of colitis and candidiasis protection is required prior to FoxP3 induction (139, 149). Therefore, the protective effects of each SCFA may require unique prerequisites which must be considered during experimental design.

#### Indole

Indole and indole-derivatives are derived from the diet or through bacterial metabolism of the amino acid tryptophan, and these molecules have diverse effects in hosts (150). Diet-derived indoles upregulate *Foxp3* and *Il10* gene expression via the activation of Aryl hydrocarbon receptors (AhR) (151–153). Moreover, indole-3-carboxaldehyde, derived from commensal bacteria or cruciferous vegetables, increased *Il10* transcripts in mice colon (154) and consequently increased the frequency and function of Tregs. Few studies, however, have examined microbially-derived indole and their derivatives on Treg function. Interestingly, butyrate indirectly ameliorated arthritis by promoting tryptophan-digesting bacteria, thereby increasing colonic levels of hydroxyindole-3-acetic acid, increasing Breg function via AhR (155). This highlights the complex interplay between bacterial metabolites and the difficulties of studying these biological products.

#### Bile Acids

Primary bile acids (BA) are synthesized by the liver, stored in the gallbladder, and secreted into the gut lumen, where they facilitate lipid digestion (156, 157). Most BAs are reabsorbed in the ileum, but 5% escape into the colon, where they undergo biotransformation by the gut microbiota. This involves deconjugation and transformation into secondary BAs, comprising mostly deoxycholic acid (DCA) and lithocholic acid (LCA) in humans (158). BAs have been shown to activate farnesoid X receptor (FXR), vitamin D receptor (VDR), and G protein-coupled bile acid receptor 1 (TGR5) (157, 159). Along with the activation of these receptors, BA metabolites may also upregulate Tregs via epigenetic and metabolic modulations.

3β-hydroxydeoxycholic acid (isoDCA) promotes pTreg differentiation by downregulating the pro-inflammatory profile of DCs (160). It was proposed that isoDCA may function via antagonism of FXR, which contradicts the consensus of BAs as FXR agonists (161). However, the authors did not show direct inhibition of FXR activity by isoDCA. In contrast, Song *et al*. found that both VDR and FXR deficiency depleted colonic RORγt^+^ Treg frequency, with VDR being the principal receptor required for BAs to maintain colonic RORγt^+^ Treg homeostasis (159). Accordingly, VDR deficiency was associated with increased disease severity in murine models of colitis; however, whether this was mediated by BAs or Vitamin D was not examined (159). In contrast, the secondary BA, isoalloLCA, could enhance pTreg differentiation by increasing H3K27 acetylation within the CNS3 region of the *Foxp3* locus (162) or by binding to the nuclear hormone receptor NR4A1 (122). Interestingly, enhancement of mitochondrial reactive oxygen species was necessary for isoalloLCA promotion of Tregs (162).

Overall, the BA-mediated induction of Tregs utilizes various mechanisms seemingly unique to each BA. Although studying the effects of individual BAs is common, this is not reflective of the diverse pool of BAs present in hosts. Indeed, Song et al. found supplementation with defined mixes of primary or secondary BAs rescued colonic Treg frequencies while singular BAs did not (159), suggesting that BA induction of Tregs likely requires synergistic activities. Unfortunately, choosing a consistent and defined pool of BAs is difficult, as differences in host diet and microbiota lead to significant individual variation in the BA pool (163).

#### Fatty Acids

Dietary fatty acids, including polyunsaturated fatty acids such as omega 3 and omega 6 can be processed by gut bacteria to a variety of metabolites. In particular, members of *Bifidobacteria* can produce conjugated linoleic acid (CLA), which increased murine Treg through PPARγ activation in a murine model of DSS-induced colorectal cancer (164). Dietary supplementation with CLA improved DSS colitis in mice by upregulating colonic IL-10, although Tregs were not directly examined (165). Linoleic acid metabolites can also inhibit Treg development with 12,13-diHOME decreasing pulmonary Tregs via the activation of PPARγ in DC, which in turn decreased their IL-10 secretion in an allergic airway model (166).

### Direct Physical Interaction or Indirect

#### Bacterial Extracellular Vesicles

The activation of host PRRs by microbial ligands present on the bacterial surface, collectively known as pathogen-associated molecular patterns (PAMPs), is a common mechanism of Treg induction. Bacterial PAMPs, such as the membrane components lipopolysaccharide (LPS) and lipoteichoic acid (LTA), can activate TLR4 and TLR2, respectively. The TLR2/MyD88 signaling pathway has been particularly well-studied in the context of Treg induction with a reduction of tTregs and pTregs in TLR2 deficient mice (167) Treg numbers were unaffected in TLR4 deficient mice (168). The most well-known commensal activation of the TLR2 pathway is by the capsular polysaccharide A (PSA) present on *Bacteroides fragilis*, inducing colonic FoxP3^+^ Tregs (130). Purified PSA promoted Treg differentiation via the activation of tolerogenic DCs (169). Whether other bacteria such as *Akkermansia muciniphila* or the defined commensal flora (known as the Altered Schaedler Flora) induce Treg via direct or indirect effects (124, 125) and through PRR activation is still unclear.

Microbial antigens can also regulate Treg biology via Treg-intrinsic signaling. Treg express TLRs, which enables their direct sensing of PAMPs. TLR engagement on Treg regulate their suppressive capacity, likely in a species-specific manner (170). TLR5 activation on human CD25^+^ Treg increased FoxP3 expression and suppressive activity *in vitro* (171). TLR2 activation on CD4^+^ T cells by *B. fragilis* PSA NCTC9342 induced Treg and augment their suppressive capacity in the absence of APCs (169). These effects are specific for PSA as the synthetic TLR2 ligand Pam3CSK4 did not affect Treg function. Another study identified that hundreds of phylogenetically diverse intestinal bacteria could produce PSA-like capsular polysaccharides (172), and lysates of these strains induced IL-10 secretion and increased CD25^+^FoxP3^+^ cells *in vitro* in human peripheral blood mononuclear cells (172). The list of commensal bacteria able to regulate host immunity is thus far from extensive.

Gut bacterial extracellular vesicles (EV) are nanosized membrane vesicles released constitutively by gram-positive and gram-negative bacteria. EV can cross the intestinal barrier under homeostatic conditions (173, 174) and disseminate to distal organs, including the liver, heart, spleen, kidney, and brain (175). The outer surface of EVs is enriched in bacterial membrane components such as LPS, LTA, peptidoglycan, and various lipoproteins. EV package inside a variety of cargo, including lipids, proteins, carbohydrates, and nucleic acid (174).

Gut bacterial EV can indirectly induce pTreg via DCs. *B. fragilis* NCTC9343 EVs induced Treg through TLR2-dependent mechanisms on tolerogenic DCs (131) as well as *Bifidobacterium bifidum* LMG13195 EVs by inducing DCs maturation *in vitro* (176). *In vivo*, a 3 day administration of *Lactobacillus rhamnosus* JB-1 EVs to mice increased proportions of IL-10^+^ DC and functionally suppressive FoxP3^+^ Treg in Peyer's patches and mesenteric lymph nodes (177). Induction of tolerogenic IL-10^+^ DC was dependent on multiple PRRs activation, including TLR2, TLR9, and the C-lectin type receptors Dectin-1 and SignR1, indicating EVs can activate multiple PRRs. *L. rhamnosus* JB-1 EVs were also enriched in heat shock proteins (HSPs) (177), a highly conserved protein family across species. HSP are critical regulators of Treg via TLR2 (178) with HSPs from *E. coli* (179), *Mycobacterium tuberculosis* (180, 181), *H. pylori* (182), as well as from helminths (183) promoting Treg *in vitro*. Treg specific for bacterial HSP can also recognize host HSP, which are released during inflammation. These Treg can as a result attenuate inflammatory disorders such as experimental arthritis (184). Furthermore, HSP-specific T cells with suppressive capacity expressed a variety Treg-associated markers such as GITR, CTLA-4, and LAG-3 (184).

Together, these studies demonstrate that EVs promote Treg through various mechanisms including via PAMPs and HSP. Other mechanisms, including small RNAs, which are important in interkingdom communication may also be involved (185).

#### Gut Bacterial DNA

Bacterial DNA contains a high frequency of unmethylated CpG dinucleotides (CpG), a ligand for TLR9. CpG content differs widely across bacteria (186) resulting in their different ability to stimulate TLR9, with the higher the frequency of unmethylated CpG nucleotides in bacterial DNA, the highest the activation of TLR9 (187). Bacterial CpG is a potent inducer of IL-10^+^ Breg *in vitro* in humans (188) and mice (189) via TLR9. In allergy, CpG-TLR9 activation on DC and B cells initiates a Th1 and Treg response that restores immune balance (190). As such, synthetic CpG oligodeoxynucleotides (ODN) have been used therapeutically in the treatment of asthma and allergy (191, 192).

Nucleic acid from gut bacteria is an essential source of TLR9 ligands critical for gut homeostasis, as apical TLR9 signaling in intestinal epithelial cells limits pro-inflammatory signals, and *Tlr*9^−/−^ mice are more susceptible to colitis (193). Oral administration of synthetic ODN (ID35) derived from *L. rhamnosus* attenuated colitis (194), and a synthetic ODN derived from *Streptococcus thermophilus* NCDO 573 (commonly found in fermented milk products) increased IL-10 expression and proportion of CD4^+^CD25^+^ Treg in murine splenocytes (195). In humans, TLR9 agonists are used as anti-inflammatory therapy for ulcerative colitis (196) and ODN-primed human pDC induced functionally suppressive CD4^+^CD25^+^ Tregs (197).

While unmethylated CpG nucleotides canonically activate TLR9, methylated bacterial DNA has recently emerged as another immunomodulatory factor. DNA isolated from a commensal strain of *Bifidobacteria longum subsp. infantis* S12 strongly induced CD25^high^FoxP3^+^ Tregs *in vitro* in a dose-dependent manner, whereas DNA isolated from *Lactobacillus rhamnosus* GG or *E. coli* strain B had significantly weaker Treg-promoting potential (198). This was attributed to a methylated CpG motif unique to the *Bifidobacteria* strain. Accordingly, a methylated CpG DNA octamer was more effective than an unmethylated octamer at converting FoxP3^+^ cells from CD4^+^ cells *in vitro* (199). GC-rich motifs are immunosuppressive motifs identified in commensal bacteria (195, 200). Administration of synthetic GC-rich suppressive sequences in mice induced Tregs and protected from DSS-colitis (200).

Overall, the gut microbiota is a rich source of nucleic acids either packaged in EVs or released as extracellular DNA. These nucleic acids have the potential to regulate intestinal Tregs, via TLR9, as well as other mechanisms yet to be identified.

## Gut Microbiota and TTREG

While the impact of the gut microbiota on the differentiation of pTreg is well-established, its impact on tTreg is unclear. GF mice have defects in lymphoid and mucosal tissues yet their thymus cellularity and development is typically reported as normal (201). The gut microbiota has recently shown to influence the T cell repertoire by bacterial antigen-specific T cells in the thymus. Bacterial antigens are presented by CX_3_${\text{CR}}_{1}^{+}$ DC that migrates from the colon to the thymus. This effect was limited to naïve T cells as tTreg were not impacted (27). While this work shows that gut bacterial antigens can be trafficked to the thymus via migratory DC, it is unknown whether bacterial components can reach the thymus via other mechanisms. tTreg are affected by PAMPs as TLR2 deficient mice had decreased tTreg and pTreg (167) and TLR9 activation in mTEC promotes the recruitment of CD14^+^ monocyte-derived DCs to the thymus, affecting the negative selection process and tTreg generation (202). Furthermore, bacterial metabolites may also influence tTreg as butyrate has been shown to increase AIRE expression in mTEC *ex vivo* and promoted tTreg in a fetal thymic organ culture model in a GPR41-dependent manner (203). Similarly, acetate increase AIRE expression in mTEC without affecting tTreg numbers (204). There are evidence showing that bacterial antigens, PAMPs and metabolites reach the thymus where they may affect tTreg development. However, the mechanisms involved, as well as the consequence of gut microbiota alteration on tTreg development remain elusive.

## Conclusions and Perspectives

There are multiple means by which Treg differentiation can be regulated by both diet and the gut microbiota. These includes epigenetic changes, alteration to T cell metabolism, and the engagement of host receptors, such as TLRs and AhR. Diet can influence other immune subsets and regulate physiological processes, such as bile acid biology, to regulate Treg biology. The concerted contribution of these pathways may be required for optimal Treg induction and the maintenance of immune tolerance. All aspects of diets (macronutrients, micronutrients, and additives) have been shown to regulate Treg biology to varying extent, suggesting that Treg development is highly responsive to the nutritional status of the host. Indeed, defects in Treg development associated with the adoption of a western diet may underlie the increasing incidence of inflammatory diseases such as autoimmunity, allergies, and inflammatory bowel disease in western countries. Accordingly, dietary intervention or symbiotic (combination of pre- and probiotics) may prove to be a viable strategy in restoring Treg numbers to prevent or treat diseases. It is not clear, however, if any one type of dietary intervention would be effective in restoring Tregs across different disease contexts. T cell development and Treg ontogeny also exhibit high plasticity throughout life, and whether a dietary intervention would be effective at promoting Treg throughout the different life stages remains to be investigated. Maternal nutrition and maternal gut microbiota can also shape the neonatal immune system (205), suggesting that dietary modulation of Treg biology likely begins as early as fetal development. Addressing these questions would allow us to better understand how interventions can be personalized for therapeutic purposes.

## Author Contributions

JTan, JTai, and LM wrote, reviewed, and edited the manuscript. DN wrote the manuscript and created the figures. SS and LL wrote the manuscript. All authors contributed to the article and approved the submitted version.

## Funding

This project was funded by the Australian Research Council grant APP160100627. DN and JTai are recipients of the Australian Government Research Training Program Scholarship.

## Conflict of Interest

The authors declare that the research was conducted in the absence of any commercial or financial relationships that could be construed as a potential conflict of interest.

## Publisher's Note

All claims expressed in this article are solely those of the authors and do not necessarily represent those of their affiliated organizations, or those of the publisher, the editors and the reviewers. Any product that may be evaluated in this article, or claim that may be made by its manufacturer, is not guaranteed or endorsed by the publisher.
